# Intratumoral immunotherapy with STING agonist, ADU-S100, induces CD8+ T-cell mediated anti-tumor immunity in an esophageal adenocarcinoma model

**DOI:** 10.18632/oncotarget.27886

**Published:** 2021-02-16

**Authors:** Ali H. Zaidi, Ronan J. Kelly, Anastasia Gorbunova, Ashten N. Omstead, Madison S. Salvitti, Ping Zheng, Juliann E. Kosovec, Soyoung Lee, Shahin Ayazi, Laila Babar, Gene G. Finley, Ajay Goel, Blair A. Jobe

**Affiliations:** ^1^Esophageal and Lung Institute, Allegheny Health Network, Pittsburgh, PA, USA; ^2^Department of Hematology and Oncology, Charles A. Sammons Cancer Center, Baylor University Medical Center, Dallas, TX, USA; ^3^Department of Radiation Oncology, Allegheny Health Network, Pittsburgh, PA, USA; ^4^Department of Molecular Diagnostics and Experimental Therapeutics, Beckman Research Institute of City of Hope, Monrovia, CA, USA; ^*^Co-first authors and contributed equally to this work

**Keywords:** esophageal adenocarcinoma, STING, PD-L1, CD8+ T-cells, IFNβ

## Abstract

Background: Esophageal adenocarcinoma (EAC) is a deadly disease with limited treatment options. STING is a transmembrane protein that activates transcription of interferon genes, resulting in stimulation of APCs and enhanced CD8+ T-cell infiltration. The present study evaluates STING agonists, alone and in combination with radiation to determine durable anticancer activity in solid tumors.

Materials and Methods: Esophagojejunostomy was performed on rats to induce reflux leading to the development of EAC. At 32 weeks post operatively, rats received intratumorally either 50 μg STING (ADU-S100) or placebo (PBS), +/– 16Gy radiation. Drug activity was evaluated by pre- and post- treatment MRI, histology, immunofluorescence and RT-PCR.

Results: Mean MRI tumor volume decreased by 30.1% and 50.8% in ADU-S100 and ADU-S100 + radiation animals and increased by 76.7% and 152.4% in placebo and placebo + radiation animals, respectively (*P* < 0.0001). Downstream gene expression, pre- to on- and post- treatment, demonstrated significant upregulation of IFNβ, TNFα, IL-6, and CCL-2 in the treatment groups vs. placebo. On- or post- treatment, radiation alone, ADU-S100 alone, and ADU-S100 + radiation groups demonstrated enhanced PD-LI expression, induced by upregulation of CD8+ T-cells (*p* < 0.01).

Conclusions: ADU-S100 +/– radiation exhibits potent antitumor activity and a promising immunomodulatory profile in a *de novo* EAC.

## INTRODUCTION

Esophageal cancer is the sixth most common cause of cancer related deaths worldwide [1]. Currently, the mainstay of treatment consists of chemotherapy, radiation, surgery, PD-1/PD-L1 checkpoint blockade in microsatellite instability high (MSI-H) tumors, and targeted therapy with monoclonal antibodies targeting HER2 [2]. Despite these therapeutic modalities, overall survival in stage IV patients remains poor with a 5 year survival rate of less than 20% [3]. Therefore, new advancements to enhance therapeutics efficacy are much needed.

Targeting the PD-1/PD-L1 axis has proven to be highly efficacious in patients with advanced solid tumors including melanoma, renal cell carcinoma, and non-small cell lung cancer [4, 5]. Durable responses to ICIs are typically observed in the presence of tumor-infiltrating T-cells (TIL) [6]. In gastroesophageal cancers PD-L1 upregulation occurs in approximately 40% of patients, predominantly on infiltrating myeloid cells at the invasive margin rather than cancer cells [7, 8]. However, initial results of ICIs in the treatment of esophageal cancer (EC) have been marginal with encouraging signals mostly limited to esophageal squamous cell carcinoma (ESCC), MSI-H, and subgroups with combined positivity score (CPS) > 10 [2]. Therefore, a future promising strategy to enhance efficacy of ICIs in non-immunogenic cold tumors, such as esophageal cancer, is to promote T-cell infiltration by activation of the innate immune system [9].

The key regulator of the innate immune system is the stimulator of interferon genes (STING), an endoplasmic reticulum adaptor protein, which stimulates the production of type 1 interferons (IFN) from cancer cells and dendritic cells (DC) in the tumor microenvironment. Briefly, the tumor or pathogen derived cytosolic DNA in these cells is recognized by cytosolic enzyme cGAMP synthase leading to the generation of cyclic GMP-AMP, which in turn binds to and activates STING signaling. This initiates a cascade where STING in the cytoplasm binds to TANK-binding kinase 1 (TBK1) and IκB kinase (IKK), that activates a host of transcription factors: interferon regulatory factor 3 (IRF3), nuclear factor-κB (NF-κB), and signal transducer and activator of transcription (STAT6). Subsequently, nuclear translocation of these transcription factors leads to the induction of type I IFNs and other immune modulatory cytokines [10, 11]. Type 1 IFNs have multiple immune-stimulatory effects that include activation, maturation, and migration of multiple immune cells including natural killer (NK) cells, DCs and anti-tumor T-cells [12].

Therefore, as expected, in preclinical models STING agonists have demonstrated not only potent activity against the targeted primary tumors but also for distant metastasis and recurrence [13, 14]. Additionally, combination of STING agonists to standard of care radiation and immunotherapy have demonstrated enhanced antitumor activity [15, 16]. Specifically, Deng et al demonstrated in mice that damaged double stranded DNA from irradiated cells leads to enhanced activity of STING pathway in DCs to promote radiation-induced type I IFN immune response [17].

In this study we evaluate the impact of a STING agonist, ADU-S100, a synthetic cyclic dinucleotide (CDN) agonist of STING, known to activate all human and mouse STINGs and induce the expression of cytokines and chemokines [18], in combination with radiation, on local tumor control and effector T-cell functionality using the modified Levrat model for esophageal adenocarcinoma (EAC). This surgical model of end-to-side esophagojejunostomy in rats causes chronic gastroduodenoesophageal reflux disease (GDER) inducing the development of *de novo* EAC through identical physiological and molecular processes that occur in humans [19, 20]. Additionally, the model offers an intact immune system that on exposure to radiation has demonstrated enhanced PD-L1 sensitization that is dose dependent and transient in nature [21]. This provides an ideal translatable EAC model to study synergistic efficacy and immunomodulation profiles on combining STING agonists with radiation.

## RESULTS

Thirty weeks after the Modified levrat surgery was performed, 85 rats were randomized to the four intervention groups: placebo (P) (*n* = 20), placebo with radiation (P+R) (*n* = 16), STING agonist (S) (*n* = 29) and STING agonist with radiation (S+R) (*n* = 20). The overall mortality rate post randomization was 20% (*n* = 17), with the S and S+R treatment cohort representing 64.7% of the total mortality (*n* = 11). Causes of mortality within the control groups (P and P+R) included respiratory infection secondary to aspiration of reflux (*n* = 2), strictures at the anastomotic site (*n* = 2), perforated stomach ulcers (*n* = 1) and unknown etiology (*n* = 1).Within the treatment groups (S and S+R), causes of mortality included respiratory infection secondary to aspiration (*n* = 6), strictures at the anastomotic site (*n* = 3), perforated stomach ulcers (*n* = 1) and unknown etiology (*n* = 1). There was no significant increase in observed mortality in the STING agonist treated animals (22.5%) when compared to the controls (16.7%) (*p* = 0.59). Sixty-eight animals completed the study, including 30 control (*P* = 18 and P+R = 12) and 38 treatment (S = 23 and S+R = 15) animals.

**Figure 1:**
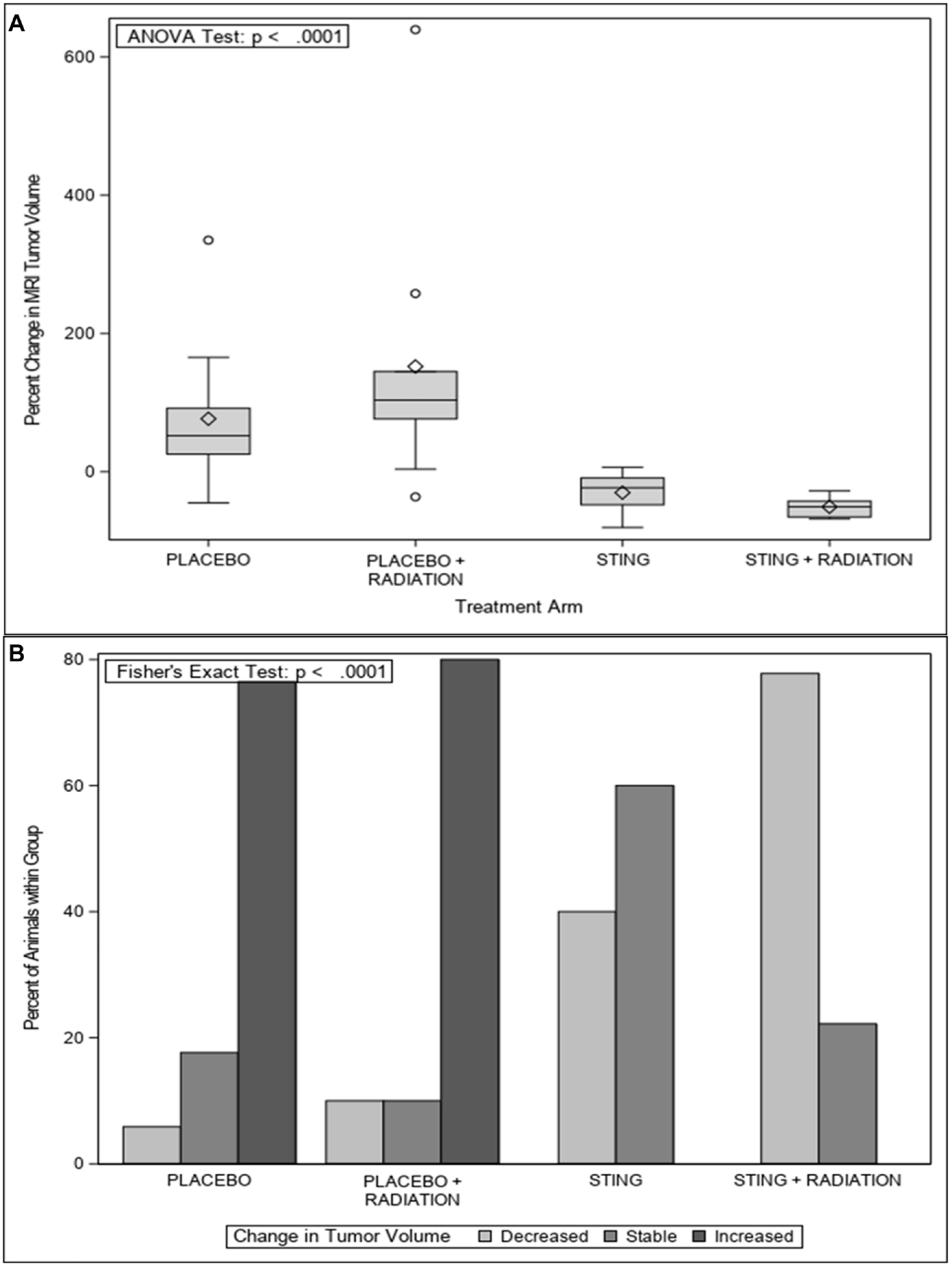
MRI change tumor volume, by treatment group. (**A**) Percent of animals with change in tumor volume as measured by MRI at 30 and 40 weeks for placebo or ADU-S100, +/– 16Gy radiation groups. (**B**) Percent change in mean tumor volume as measured by MRI from 30 to 40 weeks for placebo or ADU-S100, +/– 16Gy radiation groups.

Overall, a comparison of MRIs in the study groups between 32 and 40 weeks demonstrated a mean increase in percentage tumor volume of 76.7% and 152.4% in the P and P+R arms respectively, and a decrease of 30.1% and 50.8% in the S and S+R arms, respectively (ANOVA test *p* < 0.0001) – Figure 1A. In the P and P+R group, 76.5% and 80.0% of the rats demonstrated an increase in tumor volume, 17.6% and 10.0% had stable disease and the remaining 5.9% and 10.0% had a decrease in tumor volume, respectively. Following treatment with S and S+R, 0.0% and 0.0% of the rats demonstrated an increase in tumor volume, 60.0% and 22.2% had stable disease and the remaining 40.0% and 77.8% had a decrease in tumor volume, respectively (Fisher’s exact test *p* =< 0.0001) – Figure 1B. (Supplementary Table 1).

**Figure 2:**
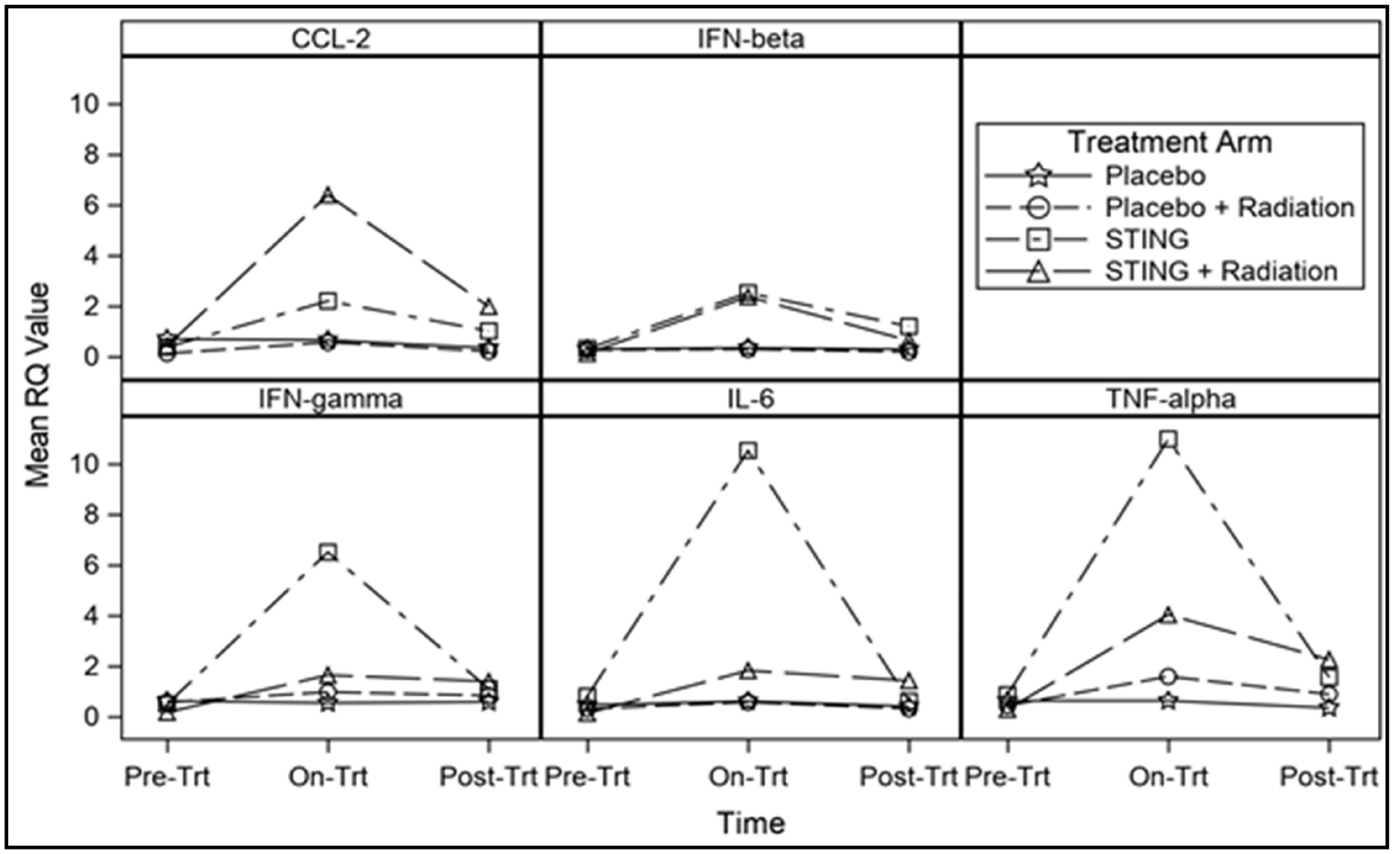
Pre and post treatment gene expression, by treatment group. Line charts demonstrating changes in mean RQ value for individual gene expression pre-, on- and post-intratumoral injections with placebo or ADU-S100, +/– 16Gy radiation. For all individual genes mixed model F-test demonstrated statistically significant difference for group effect, time effect and interaction effect between group and time, respectively (*p* < 0.01).

Analysis of STING pathway gene expression with 30-week pre-randomization biopsy RQ values set as the baseline demonstrated on- and post- S treatment a significant mean difference in RQ values. Similar but more moderate peak and trough changes in RQ values of downstream genes were observed in S+R group on- and post- treatment. On the contrary, P and P+R groups demonstrated minor difference in on- and post- treatment RQ values from baseline pre- randomization biopsy levels for all the individual genes. For the individual genes, Mixed model F-test demonstrated statistically significant difference for group effect, time effect and interaction effect between group and time, respectively (*p* < 0.01) – Figure 2. (Supplementary Table 2).

**Figure 3:**
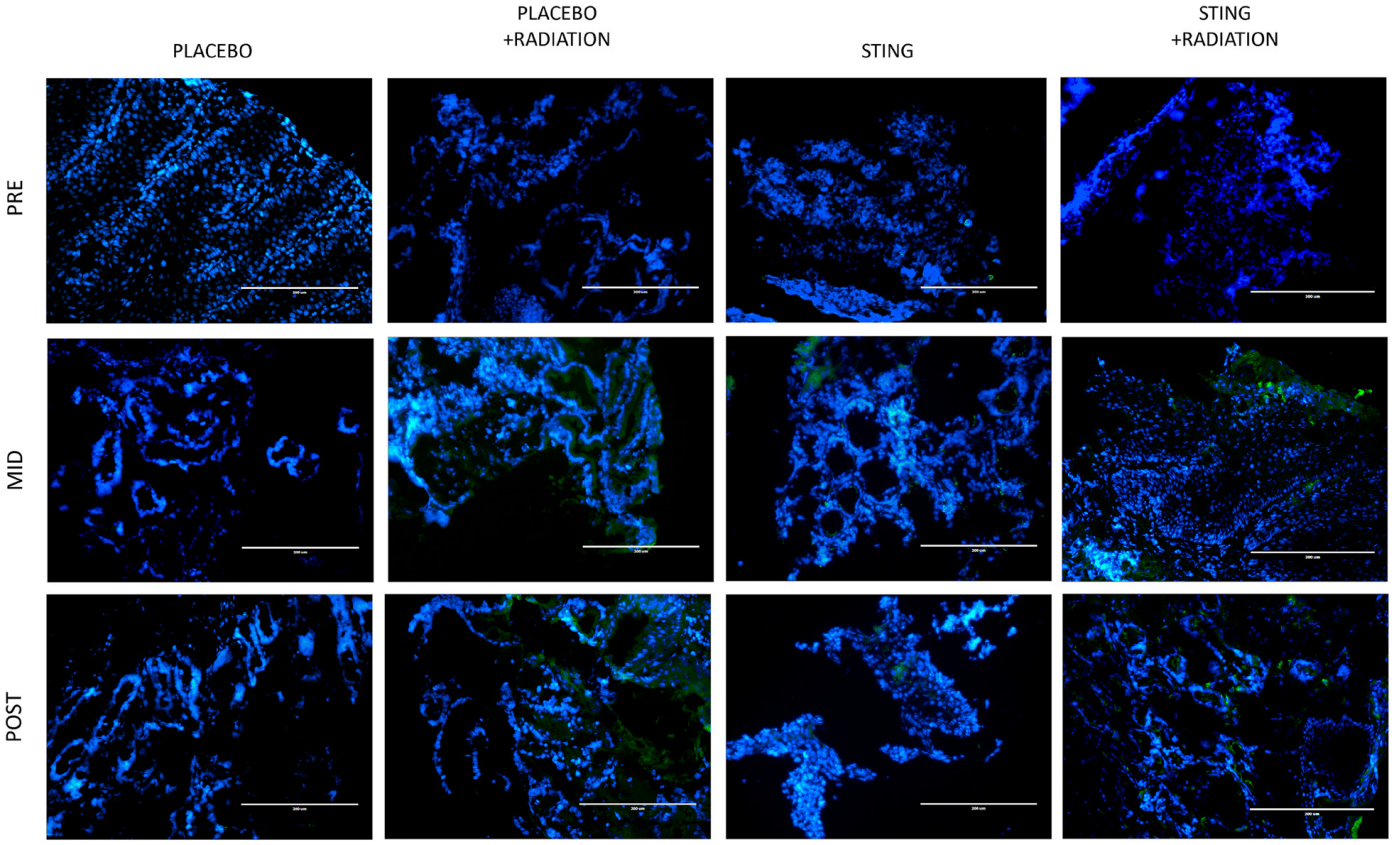
CD8 immunofluorescence, by treatment group. Representative study subgroup tumor samples showing membranous CD8 immunofluorescence pre-, on- and post-intratumoral injections with placebo or ADU-S100, +/– 16Gy radiation. Positive CD8 staining was detected at 20× magnification in the primary tumor with the Alexa Fluor 488 secondary antibody, conjugated to a green fluorophore.

**Figure 4:**
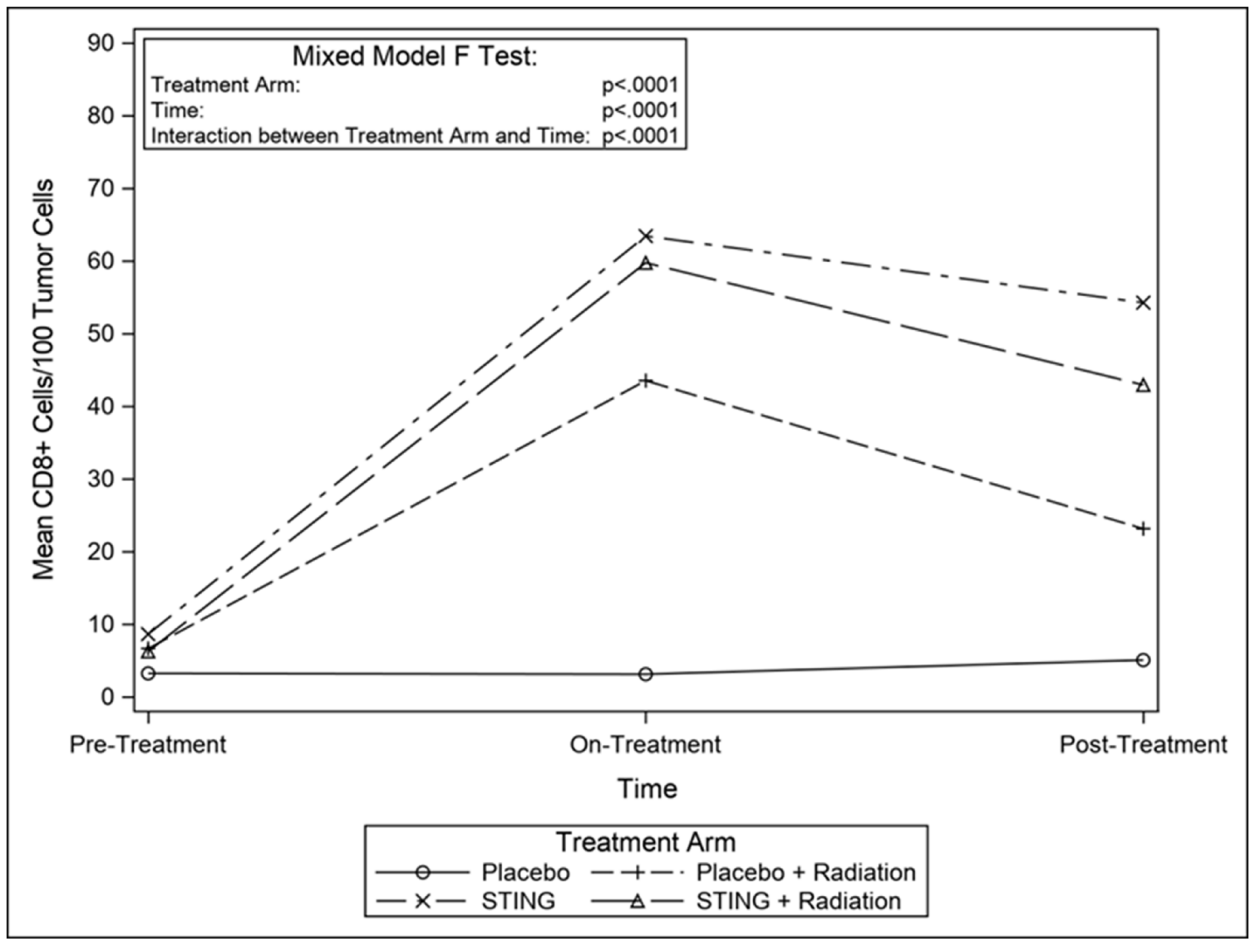
Mean CD8 cells/100 tumor cells, by treatment group. Line graphs depicting changes in mean number of CD8+ cells per 100 tumor cells by study groups pre- on- and post-intervention.

**Figure 5:**
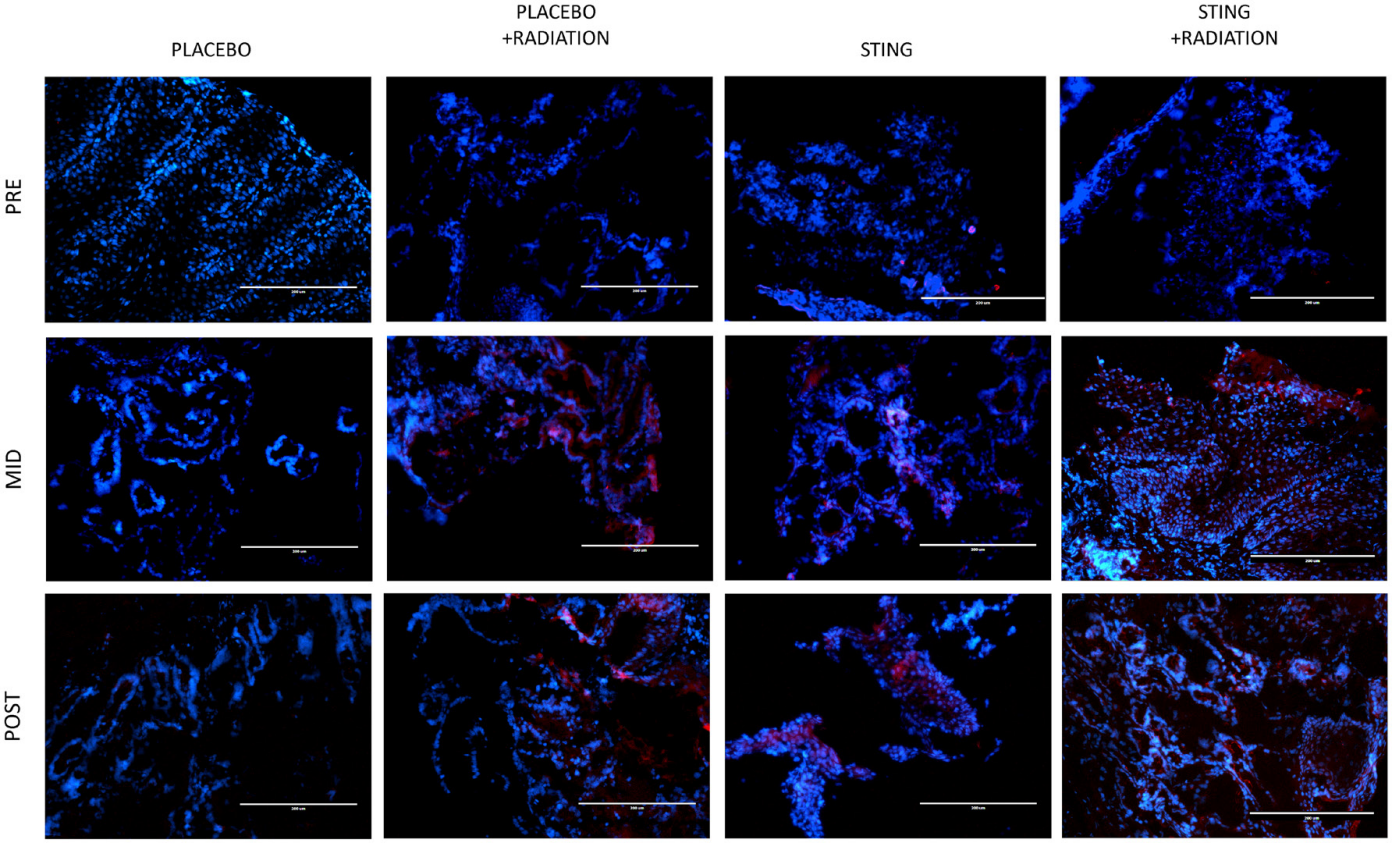
PD-L1 immunofluorescence, by treatment group. Representative study subgroup tumor samples showing membranous PD-L1 immunofluorescence pre-, on- and post-intratumoral injections with placebo or ADU-S100, +/– 16Gy radiation. Positive PD-L1 staining was detected at 20x magnification in the primary tumor with the Alexa Fluor 594 secondary antibody, conjugated to a red fluorophore.

**Figure 6:**
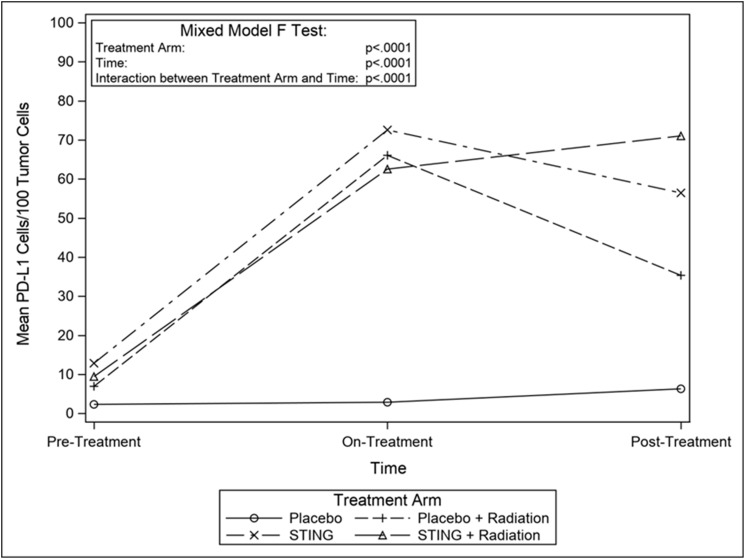
Mean CD8 cells/100 tumor cells, by treatment group. Line graphs depicting changes in mean number of PD-L1 stained cells per 100 tumor cells by study groups pre- on- and post-intervention.

TIL infiltration was upregulated with higher mean CD-8+ T-cell densities on- and post- treatment in P+R (mean on- = 43.5, *p* = 0.0009; mean post- = 23.2, *p* = 0.1044), S (mean on- = 63.4, *p* < .0001; mean post- = 54.3, *p* = 0.0010) and S+R (mean on- = 59.8, *p* = 0.0002; mean post- = 42.9, *p* = 0.0015) compared to *P* (mean on- = 3.1; mean post- = 5.1). Additionally, in the drug intervention subgroups, post-hoc analysis utilizing Wilcoxon-Mann-Whitney test showed a significant difference in CD-8 positivity between pre- and both on- (*p* < .0001 (S); *p* = 0.0002 (S+R)) and post- treatment (*p* < .0001 (S); *p* = 0.0004 (S+R)) time points – Figures 3 and 4. Subsequently, adaptive immune resistance depicted by total PD-L1 positive (immune stromal and tumor cells) was significantly enhanced in P+R (mean on- = 66.1, *p* = 0.0001; mean post- = 35.3, *p* = 0.0149), S (mean on- =72.6, *p* < .0001; mean post- =56.4, *p* = 0.0021) and S+R (mean on- = 62.5, *p* < .0001; mean post- = 71.0, *p* = 0.0015), compared to *P* (mean on- = 2.9; mean post- = 6.3). Likewise, in the drug intervention subgroups, post-hoc analysis utilizing Wilcoxon-Mann-Whitney test showed a significant difference in PD-L1 positive cells between pre- and both on- (*p* < .0001 (S); *p* = 0.0021 (S+R)) and post- treatment (*p* = 0.0003 (S); *p* = 0.0003 (S+R)) time points – Figures 5 and 6.

## DISCUSSION

Activation of the STING mechanism is central for innate immune sensing which leads to the production of pro-inflammatory cytokines, especially IFNβ, in the tumor microenvironment. IFNβ upregulation has been strongly correlated with T-cell priming and enhanced TIL densities thereby establishing the critical bridge between innate and adaptive immune responses [12, 23]. Overall, this study is the first report of a STING agonist demonstrating successful tumor regression in an EAC model while simultaneously unmasking favorable changes in the tumor immune microenvironment, such as enhanced TIL densities and PD-L1 expression. This charged immune microenvironment may provide future clinical opportunities for durable responses when combined with current ICI, chemotherapy, and radiation options [16, 17].

*In vivo*, MRI was utilized in the current study to quantify preclinical change in tumor volume in response to treatment with ADU-S100 through the comparison of 30- and 40-week scans, with each rat serving as its own control. The results demonstrated that P and P+R animals exhibited 76.7% and 152.4% increase in mean tumor volume, respectively. Meanwhile, the S and S+R treatment groups demonstrated greater than 30% reduction in mean tumor volume. Specifically, in the control arms 92.6% of the group either remained stable or increased in volume while 54.2% of the treatment animals had a reduction in volume. Overall, the S+R group demonstrated the best results with maximum mean volume reduction with all cases responding. The imaging results were further validated through gene expression and evaluation of established downstream STING pathway cytokines: IFNβ, TNFα, IL-6, and CCL2 [22]. All profiled cytokines were significantly upregulated on- and post- treatment in S and S+R groups, with the expression profiles establishing peak levels on- treatment. In the study, the *in vivo* gene expression levels validated the anticipated regulatory effects. While not evaluated in this study, evidence from other studies suggests that local tumor control is possibly mediated by IFNβ driven recruitment of cytotoxic CD8+ T-cells, TNF alpha mediated disruption of tumor microvasculature, and direct activation of apoptosis in cancer cells by STING signaling [12, 13, 23, 24].

ADU-S100 was given to S and S+R groups as 2 cycles of 50 μg intra-tumoral injections, q3 weekly. Previous data had shown this dose as immunogenic rather than tumor ablative, primarily by generating CD8+ cytotoxic T-cells for a comprehensive antitumor response through production of IFNβ via activation of STING-TBK1-IRF3 axis [25, 26]. Additionally, counterproductive vascular disruption mediated by TNF alpha for local tumor control is mostly limited at this dose selection. This is critical for generating a ‘systemic’ immune response to target distant metastasis and possible recurrences [25]. Our study in line with previous experiments successfully demonstrated enhanced PD-L1 expression, possibly by triggering an adaptive immune response, as suggested by increased infiltration of CD8+ T cells in tumors in S, S+R and P+R groups compared to P. These changes peaked on- treatment. Moreover, as reported previously, we demonstrated radiation and a STING agonist work additively in triggering an adaptive immune response [17], with S+R arm showing higher densities of IFNγ producing CD8+ T-cells when compared to radiation alone. This treatment regimen provides a significant clinical opportunity for EAC, a devastating disease with poor survival outcomes due to limited treatment options.

Overall, strategies that harness a patient’s own immune system hold great promise as 40% of gastroesophageal tumors express PD-L1, mostly on infiltrating myeloid cells [8]. Single agent PD-1 inhibitors have demonstrated modest efficacy with response rates of approximately 12% in heavily pretreated gastroesophageal cancer patients, leading to clinical approvals in metastatic 3rd line setting for pembrolizumab and nivolumab (Japan only) [27, 28]. Earlier this year, based on the data from Keynote 181 [29] and Keynote 180 [30] the Food and Drug Administration approved pembrolizumab for patients with recurrent, locally advanced, or metastatic ESCC with CPS ≥ 10, after ≥ 1 lines of systemic therapy. Although not statistically significant, better responses were observed in patients with ESCC than EAC [2, 29]. However, the most recent readout from Keynote 062 where pembrolizumab plus chemotherapy (cisplatin with either 5-fluorouracil or capecitabine) performed worse that chemo alone in untreated advanced GEJ/Gastric cancer patients dampened the excitement around the combination of PD-1 inhibitors with chemotherapy to enhance efficacy. However, the silver lining in the study was that pembrolizumab alone was deemed non-inferior to chemotherapy alone [31].

In our opinion, to improve and expand the benefit to a majority of gastroesophageal patients, beyond the response demonstrated by single agent PD-1 inhibitors, combination with other immune oncology (IO) targets is needed. Data from CheckMate 032, has demonstrated nivolumab plus ipilimumab is superior to nivolumab alone, with ORR of 24% and 12%, respectively however it is unclear if this is a viable strategy moving forwards due to enhanced toxicity with this particular IO-IO combination [32]. Currently, BMS Fraction (NCT02935634) a basket trial and Roche-Genentech Morpheus (NCT03281369) an umbrella trial are investigating multiple IO-IO or IO driven combinations in GEJ/Gastric cancers [33].

Through our current study, we provided strong evidence to suggest cross-link between the STING and PD-L1 pathways in EAC. A host of earlier preclinical cancer studies have also demonstrated enhanced activity of ICIs when combined with a STING agonist [16, 22, 34]. Currently, based on encouraging data, NCT03172936, NCT02675439 and NCT03937141 are studying ICIs in combination with ADU-S100 in human lymphomas and solid tumors.

The main limitation of our study was the inability to test a PD-1/PD-L1 inhibitor in combination with S and S+R due to unavailability of a rat cross reactive antibody. However, we did show PD-L1 upregulation on- or post- treatment with S and S+R hence substantially addressing this limitation. Another limitation may have been that we did not specifically study systemic immune response, for instance looking at peripheral T-cell trafficking. Yet previous preclinical data have well documented systemic immune modulation following a 50 μg dose of ADU-S100 [25].

In conclusion, our findings suggest potent antitumor activity of ADU-S100 alone and in combination with radiation against EAC with evident molecular pathway activation and reasonable safety. Additionally, the attractive synergistic association between STING activation and PD-L1 expression may represent a new IO-IO concurrent combinatorial antitumor strategy well-suited for further clinical testing in gastroesophageal cancers, to provide broader and more durable responses.

## MATERIALS AND METHODS

### Animal ethics statement

All animal research was performed with the approval of the Institutional Animal Care and Use Committee of Allegheny General Hospital in Pittsburgh, PA, USA, under Protocol #1057. Humane care was provided to all animals per the standards set forth in “The Guide for the Care and Use of Laboratory Animals.”

### Drug formulation and administration

Published data was utilized to select a dose strength and frequency for ADU-S100 that was deemed highly immunostimulatory [25]. ADU-S100 was provided at a concentration of 10 mg/mL by the manufacturer (Aduro Biotech Inc., Berkeley, CA, USA), diluted with PBS to a 50 μg/mL concentration, and stored at 4°C. Two cycles of a 50 μg dose of ADU-S100 or placebo (acetate buffer) were administered intratumorally q3 weekly, as 50% of the total tumor volume. Specifically, administration of the drug was accomplished using a needle through the endoscopic port of a rigid small animal endoscope [35].

### Experimental design

**Figure 7:**
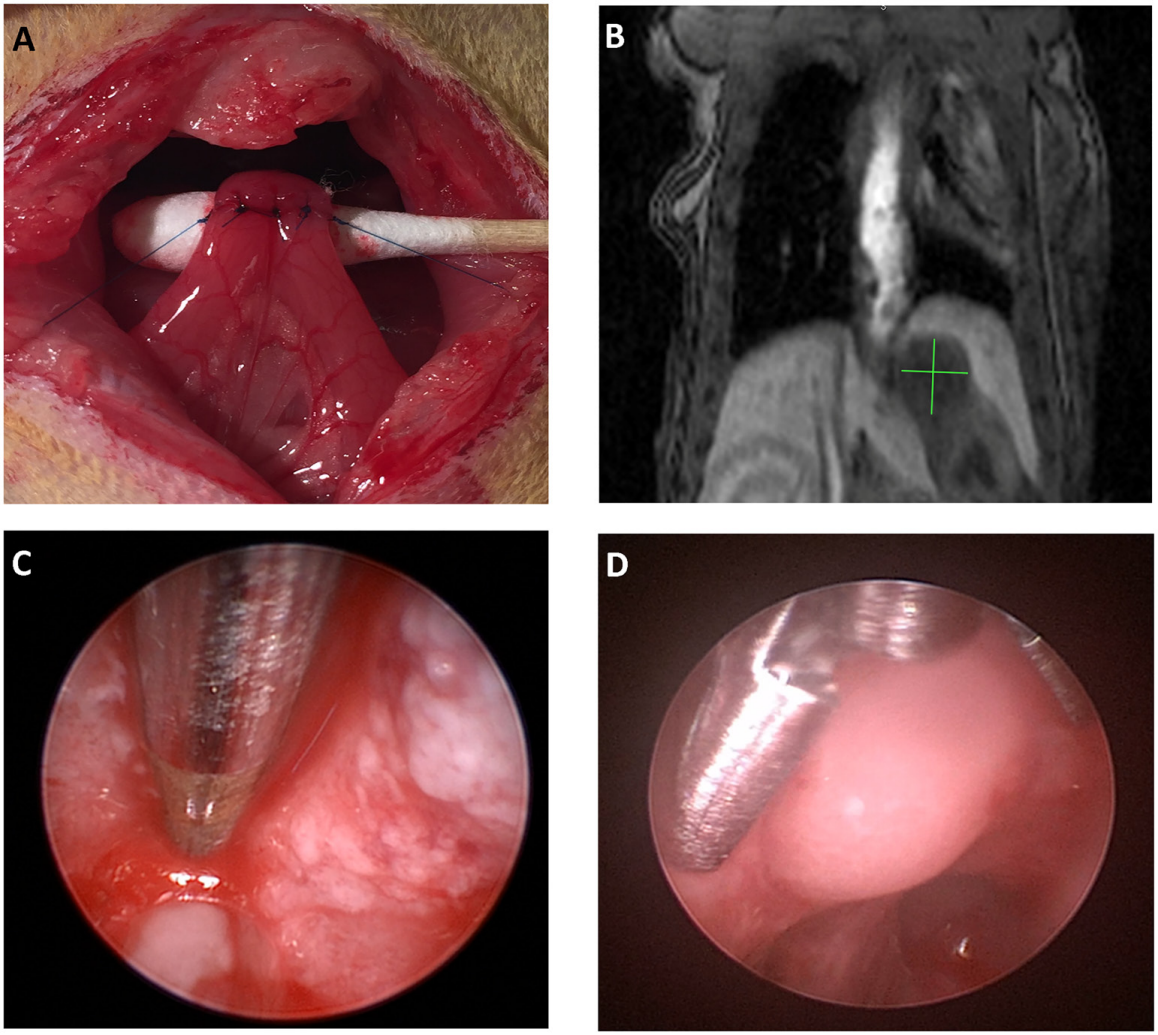
The modified levrat model. (**A**) End-to-side anastomosis of the esophagus with the jejunum performed as part of the modified Levrat surgery. (**B**) Coronal MRI image with a visible tumor at the esophagojejunal anastomosis. (**C** and **D**) Visual representation of an intratumoral injection and endoscopic biopsy of a suspected tumor in the modified Levrat model, respectively.

**Figure 8:**
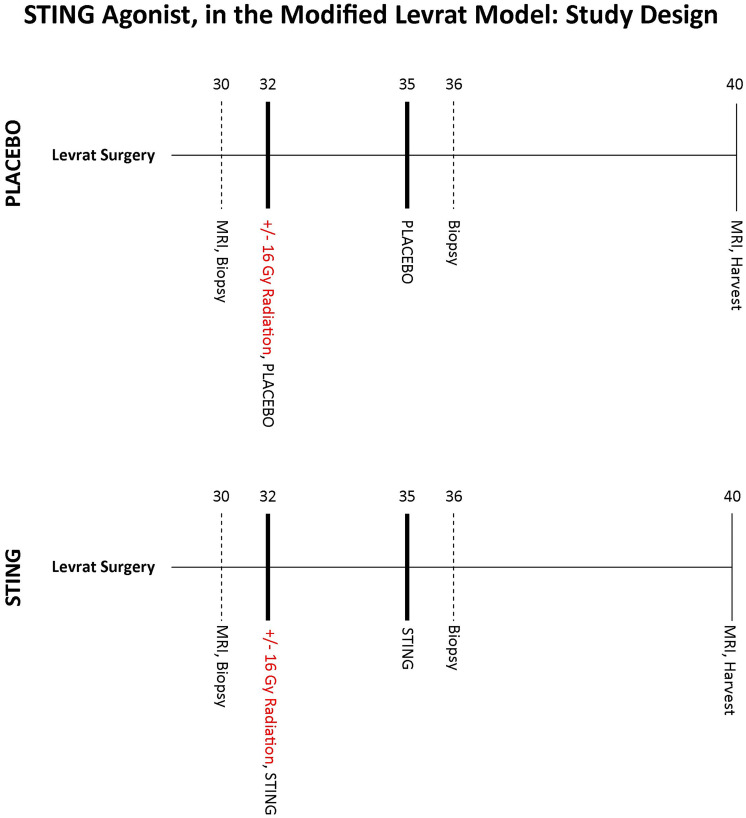
STING study design. Study design outlining the major experimental time points following randomization at 30 weeks post modified Levrat surgery. Starting at 32 weeks animals received 2 cycles of intratumoral injections of placebo or ADU-S100, q3 weekly. In addition if assigned to a radiation subgroup animals received a 16Gy dose of radiation at 32 weeks. All study rats received a final post-intervention MRI at 40 weeks followed by a final harvest of the esophagus.

Modified Levrat surgery of end-to-side esophagojejunostomy was performed on 85, 8 week old male, Sprague-Dawley rats from Harlan Laboratories, to induce GDER and progression to EAC (Figure 7A), as previously described [19] (Supplementary Figure 1). At 30 weeks post-surgery, all animals received an MRI and endoscopic biopsy of the initial primary tumor to determine initial tumor volume and baseline correlates (Figure 7B). All animals were then randomized into placebo or treatment arms (Figure 8) and if a member of a radiation group, it received one dose of 16Gy radiation at 32 weeks [21]. Placebo or ADU-S100 was administered at 32 and 35 weeks intratumorally through endoscopy at a dose of 50 μg (Figure 7C). All animals underwent a repeat biopsy at 36 weeks (Figure 7D). At 40 weeks all animals received a final MRI to determine endpoint tumor volume and were euthanized for esophageal harvest.

Activity of ADU-S100 was determined through the comparison of pre and post-treatment tumor volumes of each animal utilizing evaluable MRIs, quantitative real-time polymerase chain reaction (qRT-PCR) analysis of gene expression from serial tissue samples, and evaluation of immunofluorescence for CD8 and PDL1 proteins. MRI imaging response was scored according to the clinical gold standard of response evaluation criteria in solid tumors (RECIST version 1.1) [36].

### Tissue preparation

Pre-, on- and- post-treatment biopsy tissues were flash-frozen in Tissue-Tek Optimal Cutting Temperature (OCT) compound (Sakura Finetek, Torrance, CA, USA; #4583). Upon necropsy, the entire esophagus and jejunum, to a length approximately 1 cm distal to the anastomosis was harvested. After the specimen was cut open longitudinally, samples were rinsed in ice-cold phosphate buffered saline to remove debris, oriented to maximize exposure of suspicious areas, and flash-frozen in OCT. Next, frozen OCT blocks with adequate and evaluable tissue were cut into 5 μM sections using a cryostat (Fisher Scientific, Waltham, MA, USA; Microm HM 550) and stained with hematoxylin and eosin (H&E) for pathological assessment, gene expression analysis, and immunoassays.

### Gene expression analysis

A total of 80 μM of tissue was macrodissected using a cryostat (Fisher Scientific, Waltham, MA, USA; Microm HM 550). Briefly, RNA was isolated, reverse transcribed, and RT-PCR was performed using rat primers; CCL2 (Qiagen, Valencia, CA, USA, # PPR06714B), IFNβ (Qiagen, Valencia, CA, USA, #PPR06442B), IL6 (Qiagen, Valencia, CA, USA, # PPR06483B), IFNγ (Qiagen, Valencia, CA, USA, #PPR45050C), and TNFα (Qiagen, Valencia, CA, USA, #PPR06411F) [37]. Raw data were exported from the real-time instrument software and relative gene expression (RQ) was calculated using the ΔΔ-Ct method. Specific RT^2^ Primer Assays Endogenous controls run on each plate included SNORD-95 (Qiagen, Valencia, CA, USA, #331452), and miR16 (Qiagen, Valencia, CA, USA, #331452). All samples were normalized against pathologically confirmed normal esophagus and were run in technical duplicates.

### Immunofluorescent labeling (IF)

CD8 and PDL1 antigens were examined using an indirect double sequential immunofluorescence assay. Sample sections were fixated in 4% paraformaldehyde in PBS solution for 15 min at room temperature, then washed 3 times in 1xTBS and blocked with 1xTBS containing 5% goat serum and 1% BSA for 2 hours. Incubation with anti-CD8 antibody (Abcam, Cambridge, UK; #ab33786) was performed for 1 hour at 1:100 dilution followed by 3 TBS washes containing 0.025% Tween-20. A second incubation with anti-PDL1 antibody (Thermo Fisher Scientific, Waltham, MA, USA; #PA5-20343) at 1:100 dilution at 20 ug/mL for 1 hour was done followed by 3 TBS washes containing 0.025% Tween-20. Secondary antibody incubation was performed using 1:300 dilutions of goat anti-mouse IgG H&L Alexa Fluor 488 (Abcam, Cambridge, UK; #ab150113) and of goat anti-rabbit Alexa 594 (Thermo Fisher Scientific, Waltham, MA, USA; # A-11037) for anti-CD8 and PDL1, respectively. This was followed by 3 TBST washes. Slides were then rinsed in water and allowed to dry followed by the addition of one drop of Prolong Gold with 4′, 6-diamidino-2-phenylindole (DAPI) (Thermo Fisher Scientific, Waltham, MA, USA; #P36935). After incubation for 24 h at room temperature in the dark, cells were observed on a fluorescence microscope with light intensity 50% and exposure time of 60 ms for DAPI channel, with light intensity 60% and exposure time of 120 ms for GFP channel, and with light intensity 70% and exposure time of 750 ms for RFP channel.

For PD-L1 and CD8 staining each, a quantitative density analysis was performed for each sample using 3 selected microscopic fields and counting the number of stained cells per 100 tumor cells. Rat lymph node and PD-L1+ tumor control tissue served as positive controls for CD8 and PD-L1, respectively. Scoring was performed by three blinded trained research associates with collaborative consensus on cases with discrepant interpretation.

### Statistical analysis

An independent two-tailed *t* test or Wilcoxon-Mann-Whitney test was utilized for comparison of mean difference between any two treatment groups for CD8 cell density, PD-L1 cell density, and gene expression. ANOVA test was used to compare all groups for percent change of tumor volume. Fisher’s exact test was used for comparison of mortality status and change in MRI tumor volume among all treatment groups, respectively. A mixed model F test was performed to evaluate each gene and each protein expression between group effects (treatment group) as well as within subject effects (time point, interaction between treatment group and time point). A *p* value < 0.05 was considered to be statistically significant. All statistical analyses were performed using SAS software (version 9.4; SAS Institute, Cary, NC, USA).

## SUPPLEMENTARY MATERIALS



## Abbreviations

MSI-H: microsatellite instability high tumors
ICIs: immune checkpoint inhibitors
CAR-T: chimeric antigen receptor T-cells
PD-1: programmed cell death -1 receptor
TIL: tumor-infiltrating T-cells
EC: esophageal cancer
ESCC: esophageal squamous cell carcinoma
CPS: combined positivity score
STING: stimulator of interferon genes
IFN: interferons
DC: dendritic cells
TBK1: TANK-binding kinase 1
IKK: IκB kinase
IRF3: interferon regulatory factor 3
NF-κB: nuclear factor-κB
STAT6: signal transducer and activator of transcription
NK: natural killer cells
EAC: esophageal adenocarcinoma
GDER: gastroduodenoesophageal reflux disease
qRT-PCR: quantitative real-time polymerase chain reaction
OCT: Optimal Cutting Temperature compound
H&E: hematoxylin and eosin
RQ: relative gene expression
DAPI: Prolong Gold with 4′, 6-diamidino-2-phenylindole
P: placebo group
P+R: placebo with radiation group
S: STING group
S+R: STING with radiation group
IO: immune oncology
GEJ: gastroesophageal junction
