# Supplementary material for: Intratumoral immunotherapy with STING agonist, ADU-S100, induces CD8+ T-cell mediated anti-tumor immunity in an esophageal adenocarcinoma model
Source: Oncotarget. 2021 Feb 16;12(4):292–303. doi: 10.18632/oncotarget.27886 (PMC7899550; doi:10.18632/oncotarget.27886)
Supplement: Supplementary file 1 [file oncotarget-12-292-s001.pdf]

Intratumoral immunotherapy with STING agonist, ADU-S100, induces CD8+ T-cell mediated anti-tumor immunity in an esophageal adenocarcinoma model

SUPPLEMENTARY MATERIALS

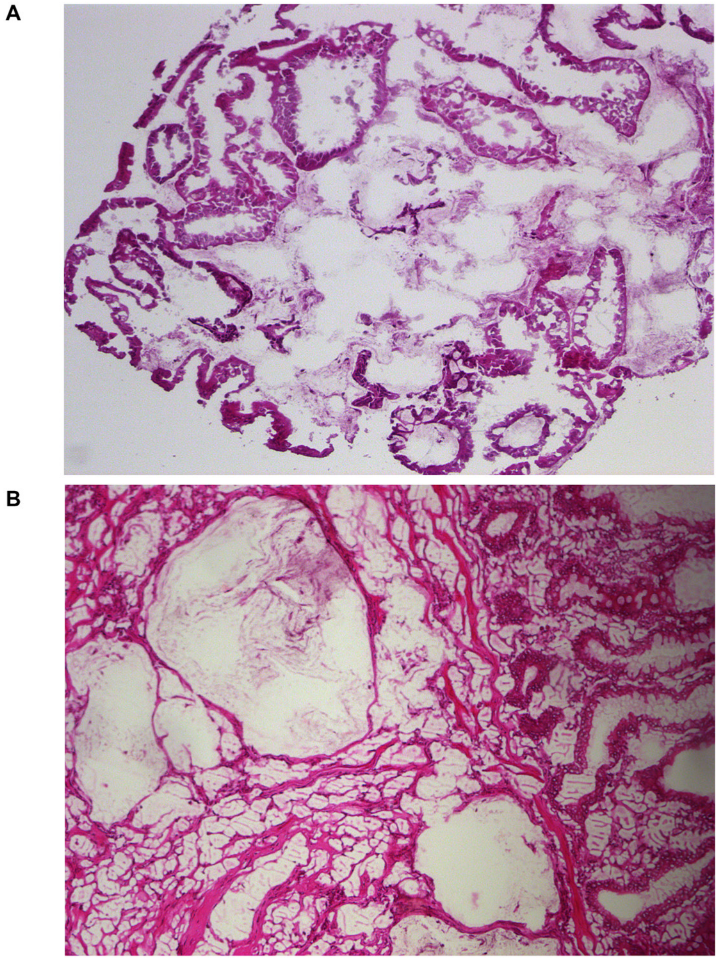

Supplementary Figure 1: (**A**) Post treatment EAC biopsy, H&E slide, 10X magnification. (**B**) Post treatment EAC endpoint distal esophagus, H&E slide, 10X magnification.

Supplementary Table 1: Change in tumor volume by treatment group

A. Placebo Group

ID	Pre/Post	Total Volume	Change in Volume (%)
**167**	Pre	8.06	134.24
**167**	Post	18.88	
**171**	Pre	9.59	165.39
**171**	Post	25.46	
**172**	Pre	4.67	6.67
**172**	Post	4.98	
**175**	Pre	6.66	82.12
**175**	Post	12.12	
**185**	Pre	12.19	10.28
**185**	Post	13.45	
**191**	Pre	5.20	163.79
**191**	Post	13.72	
**192**	Pre	4.69	92.10
**192**	Post	9.02	
**195**	Pre	12.19	76.44
**195**	Post	21.51	
**197**	Pre	15.87	45.63
**197**	Post	23.11	
**198**	Pre	7.83	25.50
**198**	Post	9.82	
**208**	Pre	4.29	78.21
**208**	Post	7.65	
**214**	Pre	13.05	38.13
**214**	Post	18.02	
**218**	Pre	9.25	15.45
**218**	Post	10.68	
**219**	Pre	6.92	27.99
**219**	Post	8.86	
**405**	Pre	10.81	52.15
**405**	Post	16.45	
**406**	Pre	18.32	335.11
**406**	Post	79.70	
**433**	Pre	9.25	-44.96
**433**	Post	5.09	

B. Placebo + Radiation Group

ID	Pre/Post	Total Volume	Change in Volume (%)
**229**	Pre	3.93	144.97
**229**	Post	9.62	
**233**	Pre	4.79	140.02
**233**	Post	11.49	

**240**	Pre	6.06	90.29
**240**	Post	11.53	
**254**	Pre	12.34	103.92
**254**	Post	25.16	
**263**	Pre	23.53	3.70
**263**	Post	24.40	
**267**	Pre	2.99	-36.26
**267**	Post	1.91	
**259**	Pre	1.44	639.70
**259**	Post	10.63	
**407**	Pre	4.16	76.50
**407**	Post	7.34	
**408**	Pre	3.88	257.88
**408**	Post	13.90	
**410**	Pre	6.35	103.39
**410**	Post	12.90	

C. STING Group

ID	Pre/Post	Total Volume	Change in Volume (%)
**282**	Pre	6.57	–1.02
**282**	Post	6.50	
**286**	Pre	7.60	–9.73
**286**	Post	6.86	
**290**	Pre	4.82	–8.98
**290**	Post	4.38	
**295**	Pre	15.06	–66.65
**295**	Post	5.02	
**303**	Pre	10.91	–23.25
**303**	Post	8.37	
**310**	Pre	5.51	6.24
**310**	Post	5.85	
**313**	Pre	98.51	–32.78
**313**	Post	66.22	
**312**	Pre	8.84	–47.73
**312**	Post	4.62	
**305**	Pre	14.48	–80.55
**305**	Post	2.82	
**411**	Pre	13.78	–68.87
**411**	Post	4.29	
**413**	Pre	32.94	–27.61
**413**	Post	23.84	
**415**	Pre	21.67	–47.37

**415**	Post	11.41	
**416**	Pre	5.82	–7.78
**416**	Post	5.37	
**418**	Pre	6.00	–19.20
**418**	Post	4.85	
**432**	Pre	170.72	–16.95
**432**	Post	141.77	

D. STING + Radiation Group

ID	Pre/Post	Total Volume	Change in Volume (%)
**201**	Pre	5.97	–50.72
**201**	Post	2.94	
**330**	Pre	3.84	–42.33
**330**	Post	2.21	
**338**	Pre	25.80	–68.35
**338**	Post	8.17	
**337**	Pre	10.51	–49.66
**337**	Post	5.29	
**423**	Pre	20.69	–65.33
**423**	Post	7.17	
**424**	Pre	8.52	–27.55
**424**	Post	6.17	
**425**	Pre	8.94	–65.97
**425**	Post	3.04	
**429**	Pre	78.12	–58.83
**429**	Post	32.16	
**430**	Pre	6.51	–28.38
**430**	Post	4.67	

Supplementary Table 2: Mean RQ values with SD by treatment group

A. IFNγ pre/on treatment difference means among arms

Treatment Arm	*N*	Mean (SD)	*P* value
Placebo	11	-0.08 (0.30)	0.0002
Placebo + Radiation	8	0.41 (0.72)	
STING	19	6.01 (5.89)	
STING + Radiation	11	1.47 (0.85)	

B. IFNγ pre/post treatment difference means among arms

Treatment Arm	*N*	Mean (SD)	*P* value
Placebo	8	0.02 (0.58)	0.0236
Placebo + Radiation	7	0.28 (0.48)	
STING	8	0.66 (0.66)	
STING + Radiation	9	1.22 (1.16)	

C. TNFα pre/on treatment difference means among arms

Treatment Arm	*N*	Mean (SD)	*P* value

Placebo	11	-0.001 (0.33)	0.0026
Placebo + Radiation	8	1.15 (0.74)	
STING	19	10.12 (11.53)	
STING + Radiation	11	3.74 (2.76)	

D. TNFα pre/post treatment difference means among arms

Treatment Arm	*N*	Mean (SD)	*P* value
Placebo	8	-0.17 (0.33)	0.0192
Placebo + Radiation	7	0.46 (0.75)	
STING	8	1.11 (1.63)	
STING + Radiation	9	1.94 (1.82)	

E. IL6 pre/on treatment difference means among arms

Treatment Arm	*N*	Mean (SD)	*P* value
Placebo	11	0.15 (0.28)	0.0092
Placebo + Radiation	8	0.27 (0.69)	
STING	19	9.73 (13.50)	
STING + Radiation	11	1.69 (1.35)	

F. IL6 pre/post treatment difference means among arms

Treatment Arm	*N*	Mean (SD)	*P* value
Placebo	8	-0.03 (0.42)	0.0066
Placebo + Radiation	7	0.0001 (0.27)	
STING	8	0.29 (0.47)	
STING + Radiation	9	1.27 (1.35)	

G. CCL2 pre/on treatment difference means among arms

Treatment Arm	*N*	Mean (SD)	*P* value
Placebo	11	-0.05 (0.37)	0.0003
Placebo + Radiation	8	0.44 (0.52)	
STING	19	1.83 (2.10)	
STING + Radiation	11	5.98 (6.18)	

H. CCL2 pre/post treatment difference means among arms

Treatment Arm	*N*	Mean (SD)	*P* value
Placebo	8	-0.31 (0.52)	<.0001
Placebo + Radiation	7	0.06 (0.33)	
STING	8	0.50 (0.97)	
STING + Radiation	9	1.59 (0.68)	

I. IFNβ pre/on treatment difference means among arms

Treatment Arm	*N*	Mean (SD)	*P* value
Placebo	11	0.04 (0.10)	0.0004
Placebo + Radiation	8	0.03 (0.45)	
STING	19	2.20 (2.01)	
STING + Radiation	11	2.24 (1.91)	

J. IFNβ pre/post treatment difference means among arms

Treatment Arm	*N*	Mean (SD)	*P* value
Placebo	8	0.05 (0.09)	0.0015
Placebo + Radiation	7	-0.08 (0.43)	
STING	8	0.85 (0.65)	
STING + Radiation	9	0.49 (0.45)	
